# The Late Meeting Texas State Medical Association

**Published:** 1888-05

**Authors:** 


F. E. DANIEL, M. D., EDITOR AND PUBLISHER.

THIS JOURNAL, by unanimous vote of the members, was made the Official Organ of the Austin District Medical Society.

COLLABORATORS:

*Dr. R. M. Swearingen., Austin.*
*Dr. B. E. Hadra, Austin.*
*Dr. Geo. Cupples, San Antonio.*
*Dr. T. C. Osborn, Cleburne, Texas.*
*Dr E J. Doering, Chicago.*
*Dr Odo Betz, Germany.*
*Dr. J. W. McLaughlin, Austin.*
*Dr. T. J. Tyner, Austin.*
*Dr. J. F. Y. Paine, Galveston.*
*Dr. E. Goldmann, San Jose, C*
*Dr. H. O. Marcy, Boston.*
*Dr. E. Meierhof, New York.*
*E. J. Beall, M. D., Fort Worth, Texas.*

### THE LATE MEETING TEXAS STATE MEDICAL ASSOCIATION.

The Twentieth Annual Convention of the Texas State Medical Association has become a matter of history. It was a success. True, the number of papers read, nor the number of physicians in attendance, nor the number of accessions to the ranks, will not compare with the last three meetings; but then, most of the papers which were read, were good, and worth preserving; and two hundred and fifty delegates in attendance, all things considered, is a fair meeting, and quite satisfactory. Galveston is on the extreme limit of the State, and it is not to be expected that there are many whose zeal in the cause will, like our esteemed friend Eastland, induce them to traverse the entire length of the State to be present. It is a matter of sincere congratulation that all passed off harmoniously and pleasantly, not a single incident occurred to mar the enjoyment of the guests, nor disturb the harmony of the meetings. There was a little friction upon the introduction of a new feature,—the attempt to imitate the American Medical Association, and have four Sections going on at once. We first advocated this plan, but the idea was suggested at the Dallas meeting, at which there were present, perhaps, four hundred and fifty, and it was found impossible to get through with the section work, so difficult was it to handle the general sessions, and keep them down to work. But, the experiment has been tried; tried at a comparatively small meeting, and it *will not do.* When the meeting was divided up into four sections, making allowance for the absence of many who preferred sight seeing, or social matters outside, the attendance at each was slim. But a worse feature is, the General Secretary cannot be present at each Section meeting, and the Section Secretaries being, as a rule, inexperienced in reporting discussions, failed in most instances to take notes of the remarks on the papers read, and hence the papers, such as are published, will appear in the transactions without the discussions, which are usually an interesting adjunct. Again, the Secretaries failed, in some instances, to turn over the papers to the General Secretary, (who is ex officio chairmon of the Publishing Committee,) and the result is, that the Publishing Committee have not yet received all the papers which were read or presented. [Some papers are in the hands of Chairman, by permission, for revision.—ED.]

We hope the effort to run four Sections simultaneously will not be repeated at any future meeting. We see that the plan (which we urged last year,) *will not do*, unless there is a large attendance and many papers.

We give credit to the worthy and most zealous Committee of Arrangements, and compliment them on the completeness of the programme orranged; but still we feel it to be our duty to point out certain defects, errors of judgment, in order to prevent a repetition. It was unfortunate to have the places of meeting of the Sections so scattered and remote from the hall, and also to have the exhibits so entirely separated, if other arrangements could have been made. We trust our new Committee of Arrangements will ponder these suggestions, and at San Antonio, next April, let all the Section meetings and the exhibits be, if possible, in one building, or very near.

The social programme was all that could have been desired, and the banquet and the toasts will be long remembered by all who had the pleasure of being present.

*Apropos*, we are in receipt of a letter from a well-known physician, (who was not present,) criticising the exclusion of the ladies (of delegates families) from the banquet. We think ourselves that, considering the fact that there was abundant room, and provision made, and the comparatively small number of ladies along with the delegates, it would have been in exceedingly good taste to have them present. But people differ on this head. At Houston and at Dallas, the ladies were included; at other points, Fort Worth notably, like at Galveston, the banquet was a spread exclusively for the members, and a few gentlemen friends, invited guests.

Of the toasts, we have not time or space to speak; suffice it, they were appropriate, and the responses were much appreciated. In looking back over the brief space of time intervening since the meeting, we feel that, upon the whole, it was good; and while there is much cause for congratulation, there is little to regret.

